# Acute Coronary Syndrome in Young Adults from Oman: Results from the Gulf Registry of Acute Coronary Events

**DOI:** 10.4103/1995-705X.76799

**Published:** 2010

**Authors:** Prashanth Panduranga, Kadhim Sulaiman, Ibrahim Al-Zakwani, Said Abdelrahman

**Affiliations:** Department of Cardiology, Royal Hospital, Muscat, Oman; 1 Department of Pharmacology and Clinical Pharmacy, College of Medicine and Health Sciences, Sultan Qaboos University, Muscat; 2 Gulf Health Research, Muscat, Oman

**Keywords:** Acute coronary syndrome, Oman, smoking, young adults

## Abstract

**Objective:**

To assess the prevalence, risk factors, presenting features, and in-hospital outcomes of acute coronary syndrome (ACS) patients ≤40 years of age from Oman.

**Methods:**

Data were analyzed from 1579 consecutive ACS patients from Oman during May, 2006 to June, 2007, as part of Gulf RACE (Registry of Acute Coronary Events). ACS patients ≤40 years of age were compared with patients >40 years of age.

**Results:**

A total of 121 (7.6%) patients were ≤40 years of age with mean age of 36 ± 4 *vs*. 61 ± 11 years in young and old adults, respectively (*P*<0.001). More men were seen in the younger age group (81 *vs*. 60%; *P*<0.001). Among all the coronary risk factors, young patients had more history of smoking (47 *vs*. 15%; *P*<0.001), obesity (72 *vs*. 58%; *P* = 0.009), and family history of coronary artery disease (CAD) (16 *vs*. 7%; *P* = 0.001). Both groups received aspirin, statins, thrombolytic therapy, and anticoagulants equally; however, younger patients received clopidogrel, glycoprotein IIb/IIIa inhibitors, β-blockers, and in-hospital coronary angiogram more. Younger patients experienced less heart failure (6 *vs*. 27%; *P*<0.001) and in-hospital mortality, especially among STEMI patients (0 *vs*. 10%; *P* = 0.037).

**Conclusions:**

Young ACS patients from Oman have different risk profile. They were treated more aggressively and their outcome was better, which is similar to other populations. However, smoking, along with obesity and family history of CAD were strong risk factors in the young Omani ACS patients. There is a need for prevention programmes to control smoking and obesity epidemic by targeting young adults in the population.

## INTRODUCTION

Acute coronary syndrome (ACS) among young adults is relatively low when compared with older population.[1] The prevalence of young patients of less than 40 to 45 years of age among ACS patients is variable depending on the population studied and generally ranges from less than 2 to 10%.[1–9] It has been observed that there is high prevalence of current smoking, hyperlipidemia, obesity, and family history of coronary artery disease (CAD) among young ACS patients and the clinical outcome in this group of ACS patients is better than older population.[1–9]

Most of the studies involving young ACS patients are reported from Western countries and presently, there is no contemporary data on the prevalence, risk factors, clinical characteristics, and outcome of such patients in the Middle-East countries and even in Oman. Thus, the Gulf Registry of Acute Coronary Events (RACE) provides a unique database to study the demographic and clinical profile of young patients with ACS in Oman. The aim of this study was to assess the prevalence, risk factors, presenting features, and in-hospital outcomes of ACS patients ≤40 years of age from Oman.

## PATIENTS AND METHODS

In this study, we analyzed data from 1579 consecutive patients from Oman enrolled in a prospective ACS registry from the Middle East (Gulf RACE). Gulf RACE is a multinational, multicentre, prospective registry of consecutive patients above 18 years of age hospitalized with the final diagnosis of ACS (unstable angina, ST-elevation myocardial infarction [STEMI], and non-STEMI) from various hospitals in six Middle Eastern countries. There were no exclusion criteria. Recruitment was done from May 8, 2006 to June 6, 2006 and from January 29, 2007 to June 29, 2007 for a period of six months. Fifteen hospitals across Oman participated. Methods of Gulf RACE have been described previously.[10] Demographic and other baseline clinical characteristics of the patients along with in-hospital management were evaluated.

Diabetes was defined as having a history of diabetes diagnosed and/or treated with medication and/or diet or fasting blood glucose 7.0 mmol/l (126 mg/dl) or greater. Hypertension was defined as having a history of hypertension diagnosed and/or treated with medication, diet, and/ or exercise, blood pressure greater than 140 mmHg systolic or 90 mmHg diastolic on at least two occasions, or as receiving any antihypertensive drug. Hyperlipidemia was defined as history of dyslipidemia diagnosed and/or treated by a physician or total cholesterol greater than 5.18 mmol/l (200 mg/dl), low-density lipoprotein greater than or equal to 3.37 mmol/l (130 mg/dl), or high-density lipoprotein <1.04 mmol/l (40 mg/dl). Current smoker was defined as a person smoking cigarettes within 1 month of index admission. A positive family history for CAD was defined as evidence of CAD in a parent, sibling, or children before 55 years of age. Obesity was defined as body mass index greater than 25 kg/m^2^. Renal impairment was defined as serum creatinine of >176.8 µmol/l (2 mg/dl).

Diagnosis of the different types of ACS and definitions of data variables and outcome parameters were based on the American College of Cardiology clinical data standards.[11] In the current study, we stratified the cohort into younger (40 years of age and younger) and older (older than 40 years of age) groups. Outcome parameters evaluated during the hospital stay included in-hospital mortality, recurrent ischemia/re-infarction, heart failure, major bleed, and stroke. Institutional review board approval was obtained.

### Statistical analysis

Descriptive statistics were used to describe the data. For categorical variables, frequencies and percentages were reported. Differences between groups were analyzed using Pearson’s χ^2^tests (or Fisher’s exact tests for cells <5). For continuous variables, means and standard deviations were presented and analyses were conducted using Student’s *t*-test. An a *priori* two-tailed level of significance was set at the 0.05 level. Statistical analyses were conducted using STATA version 11.1 (STATA Corporation, College Station, TX).

## RESULTS

A total of 1579 patients were enrolled in the study. Table 1 shows the demographic and baseline clinical characteristics of the patients. In this study, 121 (7.6%) of the patients were ≤40 years of age with mean age 36 *vs*. 61 years in young and old adults, respectively (*P*<0.001). More men were seen in the younger age group (81 *vs*. 60%; *P*<0.001). Older patients had higher frequencies of diabetes (38 *vs*. 28%; *P* = 0.033), hypertension (55 *vs*. 29%; *P*<0.001), hyperlipidemia (36 *vs*. 16%; *P*<0.001), prior angina (50 *vs*. 21%; *P*<0.001), aspirin use (50 *vs*. 17%; *P*<0.001), chronic obstructive pulmonary disease (COPD) (5.2 *vs*. 0.8%; *P* = 0.026), renal impairment (38 *vs*. 04%; *P*<0.001), and prior stroke (3.8 *vs*. 00%; *P* = 0.019), but less history of smoking, obesity, and family history of CAD, which were high among younger patients (47 *vs*. 15%; *P*<0.001, 72 *vs*. 58%; *P* = 0.009, and 16 *vs*. 7%; *P* = 0.001, respectively).

**Table 1:** Baseline clinical characteristics of young and old adults presenting with acute coronary syndrome

Characteristic	Age ≤40 (n = 121)	Age >40 (n = 1458)	*P* value
Age, mean ± SD, years	36 ± 04	61 ± 11	<0.001
Men	98(81)	555 (60)	<0.001
Diabetes mellitus	34 (28)	875 (38)	0.033
Hypertension	35 (29)	802 (55)	<0.001
Hyperlipidemia	19 (16)	525 (36)	<0.001
Current smoker	57 (47)	218 (15)	<0.001
Obesity (BMI >25 kg/m^2^)	88 (72)	846 (58)	0.009
Family history of CAD	19 (16)	102 (6)	0.001
Prior angina	25 (21)	729 (50)	<0.001
Past MI	16(13)	277 (19)	NS
Aspirin use	21 (17)	729 (50)	<0.001
COPD	01 (0.8)	76 (5.2)	0.026
Stroke	00 (0.0)	55 (3.8)	0.019
Renal impairment	05 (04)	554 (38)	<0.001
Ischemic chest pain	103(85)	1050 (72)	0.002
Atypical chest pain	13 (11)	102 (07)	NS
Dyspnea	04 (03)	204 (14)	<0.0001
Killip class I	111 (92)	1064 (73)	<0.001
Ambulance use	30 (25)	569 (39)	<0.001
Unstable angina	52 (43)	741 (51)	0.108
Non-STEMI	30 (25)	358 (25)	NS
STEMI	39 (32)	348 (24)	0.047

NS = non-significant; SD = Standard deviation; BMI = Body mass index; CAD = Coronary artery disease; MI = Myocardial infarction; COPD = Chronic obstructive pulmonary disease; STEMI = ST-elevation myocardial infarction; Percents are column percentages. All values n (%) unless specified otherwise.

When compared with older patients, younger patients were significantly more likely to have ischemic chest pain (85 *vs*. 72%; *P* = 0.002) and Killip class I (92 *vs*. 73%; *P*<0.001), but less likely to use ambulance to arrive at hospitals (25 *vs*. 39%; *P* = 0.002). More patients in the older group presented with Killip class II, III, IV (27 *vs*. 8%; *P*<0.001) and dyspnea (14 *vs*. 03%; *P*<0.001). There were no differences in the prevalence of STEMI, non-STEMI, or unstable angina among young and older patients.

**Figure 1:**
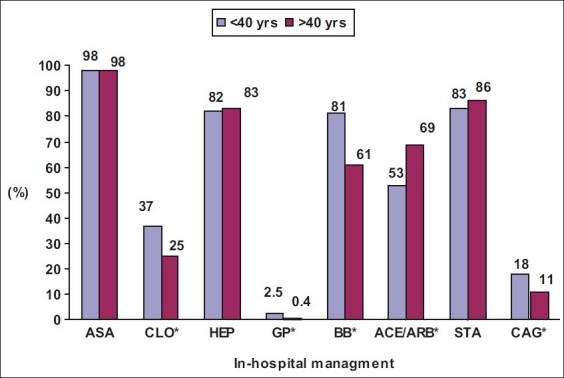
In-hospital management of young and old adults with acute coronary syndrome

**Figure 2:**
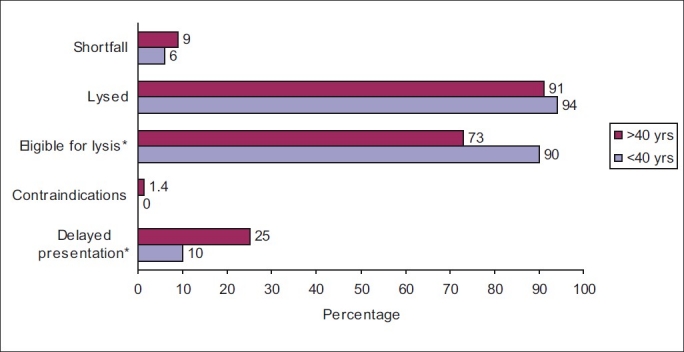
Missed opportunities for thrombolysis in young and old adults with acute coronary syndrome

Figures 1 and 2 show in-hospital management of the cohort. Both groups received aspirin, statins, thrombolytic therapy, and anticoagulants equally; however, younger patients received clopidogrel (37 *vs*. 25%; *P* = 0.003), glycoprotein IIb/IIIa inhibitors (2.5 *vs*. 0.4%; *P* = 0.026), β-blockers (81 *vs*. 61%; *P*<0.001), in-hospital coronary angiogram (18 *vs*. 11%; *P* = 0.012) more, and angiotensin-converting enzyme inhibitors (ACEIs) or angiotensin II receptor blockers (ARBs) less (53 *vs*. 69%; *P*<0.001). Figure 2 presents missed opportunities for thrombolysis in young and older adults with ACS. When compared with younger patients, older patients presented late (>12 hours) after chest pain (25 *vs*. 10%; *P* = 0.046), but there was no differences in the use of thrombolysis when indicated. Table 2 shows the complications encountered by the patients. Younger patients experienced less heart failure (6 *vs*. 27%; *P*<0.001) and significantly less in-hospital mortality, especially among STEMI patients (0 *vs*. 10%; *P* = 0.037).

**Table 2:** In-hospital outcome in young and old adults presenting with acute coronary syndrome

Characteristic	Age ≤40 (n = 121)	Age >40 (n = 1458)	*P* value
Recurrent ischemia	09 (7.4)	143 (9.8)	NS
Reinfarction	04 (3.3)	33 (2.3)	NS
Congestive heart failure	07(06)	394 (27)	<0.001
Major bleed	00 (00)	17 (1.2)	NS
Stroke	00 (00)	16 (1.1)	NS
Mortality	01 (0.8)	63 (4.3)	0.059
STEMI (n = 387)	0 (0)	36 (10)	0.037
Non-STEMI (n = 388)	1 (3.3)	13 (3.6)	NS
UA (n = 793)	0 (0)	14 (1.9)	NS

NS = nonsignificant; STEMI = ST-elevation myocardial infarction; UA = Unstable angina. Percents are column percentages. All values n (%) unless specified.

## DISCUSSION

In this study, 7.6% of patients with ACS in Oman were ≤40 years of age. In the Global Registry of Acute Coronary Events (GRACE) study, the prevalence of young ACS patients was 6.3%;[4] in the Thai ACS Registry, it was 5.8%;[5] and in the Spain registry, it was 7%.[6] This study shows that ACS in young patients occurs predominantly in men and this has also been noted in majority of populations,[1–9] suggesting that women are protected from developing ACS until menopause. Older patients had higher frequencies of multiple risk factors compared with younger patients, except for smoking, obesity, and family history of CAD. The high incidences of smoking, obesity, and family history in the young ACS patients in Oman are consistent with the results in previous reports,[1–9] except for hyperlipidemia which was low among young adults in Oman.

Smoking is an established and a predominant major risk factor in young ACS patients, being reported between 70 and 90% in the previous studies.[1–9] Smoking is known to cause increased fibrinogen concentrations and platelet aggregability, along with impaired fibrinolytic activity, decreased coronary flow reserve, and increased vasospasm.[12,13] Recurrent exposure to cigarettes with subsequent catecholamine surges damage endothelial cells, leading to endothelial dysfunction and injury of the vascular intima. Autopsy studies in young adults have showed that the extent of fatty-streak lesions in the coronary arteries of young adults was higher in smokers than in nonsmokers.[14] Even though smoking was the predominant risk factor in young ACS patients from Oman, its prevalence was low at 47% when compared with other population. Although this discrepancy cannot be explained precisely, it is possible that cultural and gender factors may be involved.

In a study, patients with a positive family history developed their first acute myocardial infarction more than 1 decade earlier in comparison with those without such a history and they were more often male smokers with less frequency of heart failure, as observed in this study.[15]

It has been documented that children born of parents with premature CAD tend to have more lipid abnormalities, insulin resistance, and obesity, strengthening the belief of a common genetic linkage and genetic polymorphisms,[14] indicating that family history of CAD is an important risk factor in young adults with ACS. Interestingly, hyperlipidemia was low in young adults with ACS from Oman. This has been noted in previous studies.[16,17] Jee *et al*.[16] noted similar rates of cardiac events in young adults with ACS between the low-cholesterol and high-cholesterol groups to mean that low cholesterol confers no benefits against smoking-related atherosclerotic cardiovascular disease.

In agreement with previous reports,[1–9] the present study has also shown that older patients had an increased frequency of poor prognostic factors like diabetes, hypertension, prior angina, aspirin use, COPD, renal impairment, and prior stroke. It is well-known that, unlike older patients, approximately half of young patients have single-vessel CAD, and in up to 20%, the cause is either nonatherosclerotic or have normal coronary arteries.[7,18,19] Nonatheromatous CAD, hypercoagulable states, hyperviscosity, and substance abuse, especially cocaine, are other important etiologies.[18] In a study, C-reactive protein was significantly lower in the young age group, with predominant single-vessel disease, whereas in older groups, it correlated positively with severity of CAD.[20] Emotional stress and hostility in young adults are thought to be related to the prevalence of CAD. In a study, the ‘hostility index’ score was directly proportional to the presence of coronary artery calcification, indicating subclinical atherosclerosis.[21] Considering higher prevalence of smoking and the higher frequency of single vessel CAD disease into consideration, coronary occlusion in young patients might be predominantly thrombogenic and vasospastic and less atherosclerotic, and therefore interventions to reduce smoking might be more effective in young adults in preventing ACS.[9] Furthermore, smoking patients with ACS may respond better to percutaneous coronary intervention or antithrombotic agents, with subsequent less significant residual stenosis and better myocardial function with good clinical outcome, thus explaining the ‘smokers paradox.’[9]

In the present study, younger patients were significantly more likely to have ischemic chest pain and Killip class I, but less likely to use ambulance to arrive at hospitals. This indicates that young adults with typical chest pain without significant heart failure tend to come directly to hospital early.[1] This is in contrast to older adults. Although older adults used ambulance more to arrive at hospitals in Oman, there was delayed presentation in STEMI patients, which may be either due to increasing frequency of atypical clinical presentation, cognitive impairment, or the presence of co-morbidities that can mask diagnosis of ACS.[4]

In the previous studies,[4,5,19,22] STEMI was more frequent in the young patients, whereas non-STEMI was in the elderly. However, in this study, there were no differences between the types of ACS among young and old adults presenting with ACS. These findings suggest that the pathophysiology in these two types of ACS may not be that different in young adults.

In the present study, both groups received aspirin, statins, thrombolytic therapy, and anticoagulants equally, indicating that evidence-based therapies were prescribed adequately. However, younger patients received clopidogrel, glycoprotein IIb/IIIa inhibitors, β-blockers, and in-hospital coronary angiogram more, indicating an aggressive approach toward young adults with ACS or presence of contraindications in the elderly. This bias has been noted in other studies as well.[4,5,22] In this study, younger adults with ACS received ACEIs or ARBs less when compared with older adults, probably because of the lower prevalence of previous cardiovascular disease, diabetes, and heart failure during hospitalization.

Previous studies have indicated that the rate of in-hospital complications and mortality are lower in young ACS patients due to low-risk profile, less severe CAD, treatment with standard therapies, and with better response to percutaneous coronary intervention.[1–9,23] In the present study, heart failure was significantly low in younger group when compared with older group. Overall, in-hospital mortality was low but was not statistically significant, likely due to small sample size. Although short-term outcome is good in young ACS patients, few reports have demonstrated that the long-term prognosis may not be benign, especially when other coexisting variables like low ejection fraction, previous myocardial infarction, previous bypass surgery, or peripheral artery disease are present.[24,25] In addition, persistence of smoking after first ACS is the most powerful predictor for the recurrence of cardiac events in patients with premature myocardial infarction.[26]

These short- and long-term data have led to a hypothesis that there exist two subgroups of young CAD patients.[27] One group with a single culprit lesion with subsequent plaque rupture on a nonsignificant vulnerable plaque precipitated by acute physical and/or emotional stress resulting in enhanced coronary shear forces is usually the mechanism of acute presentation. This group has a substantial vasospastic component superimposed on a genetic predisposition to vulnerable plaque production. Conversely, a second group is comprised of those with diabetes and others who present with established multivessel disease, with same prognosis as older patients.[27]

### Study limitations

The major limitation of our study is its observational and retrospective analysis of a prospective registry and the possible confounding variables not controlled for in the study. We report only in-hospital outcome, which may be inadequate to assess the true burden of premature coronary disease in Oman. We evaluated the standard coronary risk factors; other newer risk factors such as lipoprotein abnormalities, hypercoagable states, and elevated homocysteine levels were not studied. Furthermore, non-atherosclerotic coronary disease, which should be considered in young patients with ACS, was not evaluated.

## CONCLUSION

Oman has a significant young ACS population with a mean age of 36 years. They had different risk profile, were treated more aggressively, and their outcome was better, which is similar to other populations. Nearly half of young ACS patients were smokers, which was relatively low compared with other countries. However, smoking along with obesity and family history of CAD were strong risk factors in the young Omani ACS patients. There is a need for prevention programs to control smoking and obesity epidemic by targeting young adults in the population.
